# Synchronization of non-smooth chaotic systems via an improved reservoir computing

**DOI:** 10.1038/s41598-023-50690-4

**Published:** 2024-01-02

**Authors:** Guyue Wu, Longkun Tang, Jianli Liang

**Affiliations:** School of Mathematical Science, Huaqiao University, Quanzhou, 362021 China

**Keywords:** Complex networks, Applied mathematics

## Abstract

The reservoir computing (RC) is increasingly used to learn the synchronization behavior of chaotic systems as well as the dynamical behavior of complex systems, but it is scarcely applied in studying synchronization of non-smooth chaotic systems likely due to its complexity leading to the unimpressive effect. Here proposes a simulated annealing-based differential evolution (SADE) algorithm for the optimal parameter selection in the reservoir, and constructs an improved RC model for synchronization, which can work well not only for non-smooth chaotic systems but for smooth ones. Extensive simulations show that the trained RC model with optimal parameters has far longer prediction time than those with empirical and random parameters. More importantly, the well-trained RC system can be well synchronized to its original chaotic system as well as its replicate RC system via one shared signal, whereas the traditional RC system with empirical or random parameters fails for some chaotic systems, particularly for some non-smooth chaotic systems.

## Introduction

Chaotic systems have been widely used to many fields^1–4^, such as secure communication, image encryption, electronic circuits and so on^5,6^, due to the excellent dynamical characteristic. Since the 1990s, investigations on theory and its applications of chaotic systems have being increasingly paid attention to by lots of researchers, including on the prediction of chaotic time series as well as on synchronization between chaotic systems, and there emerge a large number of publications^7–12^.

In recent years, the great success of machine learning in artificial intelligence stirs up new research on the prediction of chaotic systems and their dynamical behaviors. For example, some tranditional machine learning methods, such as Recurrent Neural Network (RNN)^13^, Long Short-Term Memory (LSTM)^14^, Temporal Convolutional Network (TCN)^15^ and Manifold Learning^16^, are applied to predict the chaotic time series, respectively. Also, some hybrid machine learning methods^17,18^ are developed to deal with the prediction of chaotic time series, where the hybrid machine learning model proposed in the article^17^ is based on the switching mechanism set by Markov chain, and the other hybrid scheme^18^ proposed for a long-time and high-accuracy prediction is based on knowledge model and machine learning technique.

Among machine learning methods, reservoir computing (RC) method^19,20^ also called Echo State Network (ESN) and Liquid State Machine (LSM) by Jaeger^21^ and Maass^22^ respectively, greatly attracts the attention of researchers due to its superior learning and prediction ability, and has been efficiently applied to the prediction of phase coherence^23^, speech recognition^24^, extreme event^25^, and other dynamical behaviors. Furtherly, some improved methods^26–30^ of RC are also proposed for enhancing the ability in forecasting. For instance, Haluszczynski et al.^27^ improve the prediction performance of RC via reducing network size of the reservoir. Gao et al.^29^ modify RC method by preprocessing the input data via empirical wavelet transforms. Gauthier et al.^28^ develop a novel RC with less training meta-parameters (called the next generation reservoir computing) by using the nonlinear vector autoregression on the basis of the equivalence between the reservoir computing and the nonlinear vector autoregression. But, it is still difficult on the whole to develop a general RC method for data from nonlinear systems, particularly from chaotic systems.

Very recently, researchers devote themselves to the prediction of complex dynamical behaviors (such as, synchronization and chimera state) of chaotic systems by using RC method, and have published a few publications^31–36^. At the aspect of chimera state (a coexistence of coherent and non-coherent phases in coupled dynamical systems), Ganaie et al.^31^ propose a distinctive approach using machine learning techniques to characterize different dynamical phases and identify chimera states from a given spatial profile. Kushwahaet et al.^32^ use different supervised machine learning algorithms to make model-free predictions of factors characterizing the strength of chimeras and isolated states.

At the aspect of synchronization, Ibáñez-Soria et al.^36^ make use of RC to successfully detect generalized synchronization between two coupled Rössler systems. Guo et al.^33^ investigate the transfer learning of chaotic systems via RC method from the perspective of synchronization-based state inference. Weng et al.^34^ adopt RC model to reconstruct chaotic attractors and achieve synchronization and cascade synchronization between well-trained chaotic systems by sharing one common signal. Hu et al.^35^ train chaotic attractors via RC method, and realize synchronization between linearly coupled reservoir computers by control the coupling strength.

**Figure 1:**
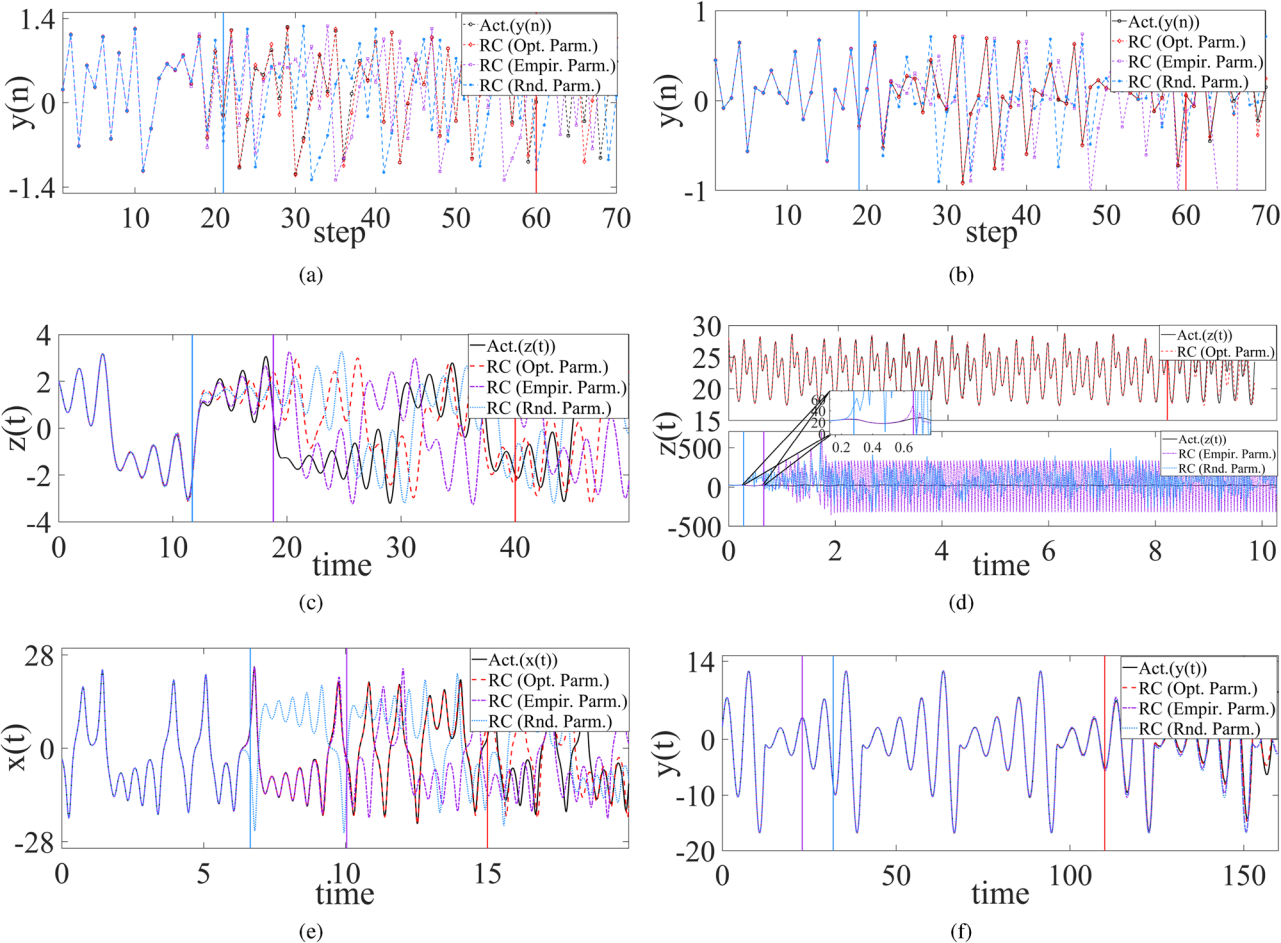
Comparison of model-free prediction effect between optimal parameters (red dashed line) based on SADE algorithm, empirical parameters (purple dash-dot line) and random parameters (cyan dotted line) for Hénon (**a**), Ikeda (**b**), Chua (**c**), PLUC (**d**), Lorenz (**e**) and Rössler (**f**) chaotic systems. Here the cyan, purple, red solid vertical lines represent the prediction time (or step) span respectively for random, empirical and optimal parameters at the same square mean error listed in Table 1.

However, all above mentioned works aim at smooth chaotic systems, few investigations involve in the prediction and learning of non-smooth chaotic systems whose dynamical behaviors may be more complicated than the smooth ones since the non-differential or discontinuous vector field in non-smooth systems easily lead to the singularity. No coincidentally, our extensive simulations show that the traditional RC method has poor prediction effect for some non-smooth chaotic time series, especially for discrete-time chaotic systems, please see Table 1 and Fig. 1. On the other hand, non-smooth chaotic systems have many applications in engineering fields, and they are frequently used to model circuit systems, such as Chua circuit system. Motivated by the above aspects, the paper proposes a hybrid RC method with optimal parameter selection algorithm for improving the prediction ability for non-smooth as well as smooth chaotic systems, and based on the improved RC model we carry out synchronization between well-trained RC systems via the variable substitution control.

## Results

In this section, based on the improved Reservoir Computing (RC) machine learning model, we will use chaotic data to check that the proposed RC model with SADE algorithm can work well for the non-smooth chaotic systems as well as the smooth chaotic systems, and to verify the availability of PC synchronization between the constructed and trained RC systems.To begin with, the fourth-order Runge-Kutta method is used to calculate numerical solutions of smooth chaotic systems (such as, Lorenz and Rössler systems shown in^37,38^) and piecewise smooth chaotic systems (such as Chua and PLUC systems), the observation time step $\Delta t=0.02$, and the numerical solution is transformed into the data at the range of $[-1,1]$ for convenience. For all the used chaotic systems, the first 3000 observations are used to train the RC system after discarding the leading appropriate amount of observations to eliminate transient states, and the first 100 observations of the trained RC system are discarded for the steady running.

**Table 1:** A list of the prediction time duration of RC systems for different chaotic systems at the given precision threshold.

Systems	*N*	*p*	*r*	Precision threshold	Duration (or steps)	Units of Lyapunov time
Hénon	235	0.0469	0.6088	$5\times 10^{-3}$	$60\,(21^{*}, 21^{\#})$	$25\,(9^{*}, 9^{\#})$
Ikeda	544	0.0173	0.1426	$5\times 10^{-5}$	$60\,(19^{*}, 19^{\#})$	$8\,(3^{*}, 3^{\#})$
Chua	274	0.0454	0.1629	$5\times 10^{-5}$	$40\,(18.78^{*}, 11.66^{\#})$	$13\,(6^{*}, 4^{\#})$
PLUC	147	0.0421	0.1993	$5\times 10^{-3}$	$20\,(0.66^{*}, 0.28^{\#})$	$31\,(1^{*}, 0^{\#})$
Lorenz	606	0.0115	0.9207	$5\times 10^{-4}$	$15\,(10.04^{*}, 6.64^{\#})$	$20\,(13^{*}, 9^{\#})$
Rössler	734	0.0411	0.5876	$5\times 10^{-8}$	$110\,(22.96^{*},31.88^{\#})$	$9\,(2^{*},3^{\#})$

Here, symbols $*$ and $\#$ represents the prediction time using the empirical and randomly-selected parameters, respectively. Lyapunov time is defined as the reciprocal of the largest Lyapunov exponent of a system. Each prediction duration of improved RC systems averages 50 realizations in the SADE algorithm, and the listed optimally-selected parameters are one of 50 experiments.

Furthermore, the basic parameters of RC model are set as follows. In Eq. 9, $\sigma =1$ for which the weight $W_{in}$ is randomly selected from the uniform distribution of $(-\sigma ,\sigma )$, the leakage rate of the reservoir is $\alpha =0.2$, and $\lambda =1\times 10^{-8}$ in Eq. 10.

### Main parameters selection and model-free prediction

This part will employ SADE algorithm mentioned in the previous section to select the appropriate parameters (*N*, *p*, *r*) for sparse Erdös-Rényi (ER) random network of RC systems, make model-free prediction of the trained RC system with the selected parameters, and compare prediction effect with those with empirical parameters as well as randomly-selected one.

Based on the presented SADE algorithm, the selected parameters in ER random networks and the prediction duration of RC for different chaotic systems are listed in Table 1, where each in 50 experiments has the same prediction duration for the improved RC at the given precision threshold. It can be seen that for PLUC chaotic system, the corresponding trained RC system can well predict up to 20 seconds (about 31 units of Lyapunov time) at the square mean prediction error of least $10^{-3}$ order. But for smooth chaotic systems, Lorenz and Rössler systems, surprisingly, the trained RC for Rössler system can predict up to 110 seconds (about 9 units of Lyapunov time) at the prediction error of least $10^{-8}$ order. Similarly, the trained RC also performed well for Lorenz system, and the prediction time is up to 20 units of Lyapunov time at the error of $10^{-4}$ order, more 7 and 11 units than the empirical and randomly-selected parameter approaches, respectively. For the discrete Hénon and Ikeda chaotic systems, the trained RC can predict up to 60 steps (about 25 and 8 units of Lyapunov time, respectively) at the prediction error of least $10^{-3}$ order and $10^{-5}$ order, respectively.

More importantly, for all the considered chaotic systems including discrete, piecewise smooth, and smooth chaotic systems, the prediction time duration of RC with optimally-selected parameters is far longer at the same of prediction precision threshold than those with empirical parameters ($N=500$, $p=0.25$ and $r=0.95$) as well as those with randomly-selected parameters, see the last two columns in Table 1, and the corresponding state variable evolution shown in Fig. 1.

**Figure 2:**
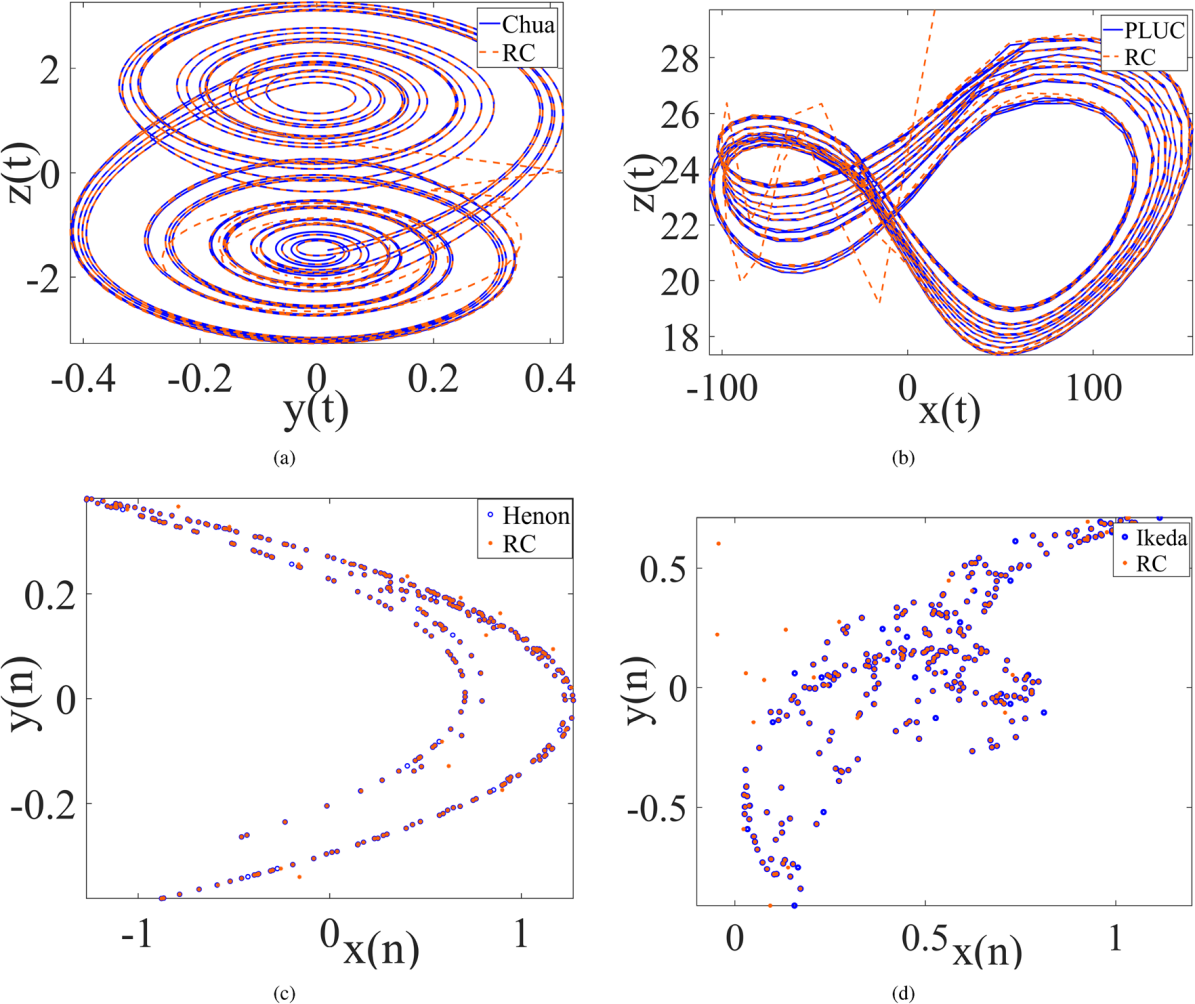
The phase diagram of synchronization state evolution between the chaotic drive system and its trained RC response system where *x* (*y*) component is the drive variable in **a**,**c**,**d** (**b**). (**a**) Chua system, (**b**) PULC system, (**c**) Hénon system, (**d**) Ikeda system.

Specifically, the Lorenz-trained RC system with optimal parameters can predict up to 15-seconds time span, about 20 units of Lyapunov time, at the error of $5.67\times 10^{-4}$, but that with empirical parameters (random parameters) just can predict up to about 10 (6.6)-seconds time span, about 13 (9) units of Lyapunov time, at the same error. Surprisingly, for another smooth chaotic system, Rössler system, the trained RC system with optimal parameters can reach up to 110-seconds prediction time span, about 9 units of Lyapunov time, with high accuracy ($8.89\times 10^{-8}$ order error), but those with empirical parameters and with randomly-selected parameters has much less prediction time at the same of error, 22.96 seconds and 31.88 seconds (about 2 and 3 units of Lyapunov time), respectively. Please see Fig. 1e,f for the state evolution.

Particularly for non-smooth chaotic systems, the trained RC systems with optimal parameters have the overwhelming prediction effect. For example, at the same error, the Chua-trained RC system with optimal parameters has 40-seconds prediction time, far bigger than those with empirical parameters (18.78-seconds prediction time) and with randomly-selected parameters (11.66-seconds prediction time), as shown in Fig. 1c. For PLUC system, there are huge difference of prediction effect between using optimal parameters and using empirical (or randomly-selected) parameters, the former reaches up to 20-seconds prediction time at the error listed in Table 1, and the latter just predicts less than 1-second time span at the same error, as shown in Fig. 1d. For discrete Hénon and Ikeda chaotic systems, their trained RC systems with optimal parameters can predict up to 60 steps, three time as much as those with empirical (or randomly-selected) parameters where both have the same prediction steps, exactly as cyan and purple vertical lines overlap each other in Fig. 1a,b.

In brief, compared with the traditional RC systems (using empirical or randomly-selected parameters), the improved RC systems based on SADE parameter selection algorithm can make a far better prediction not only for non-smooth chaotic systems, but for smooth chaotic systems (such as Lorenz system and Rössler system). In other words, the improved RC system can better learn the chaotic systems including smooth and non-smooth ones. In next section, the advantage will be shown again for PC synchronization behaviors between chaotic system and its trained RC system as well as between two trained RC systems.

**Figure 3:**
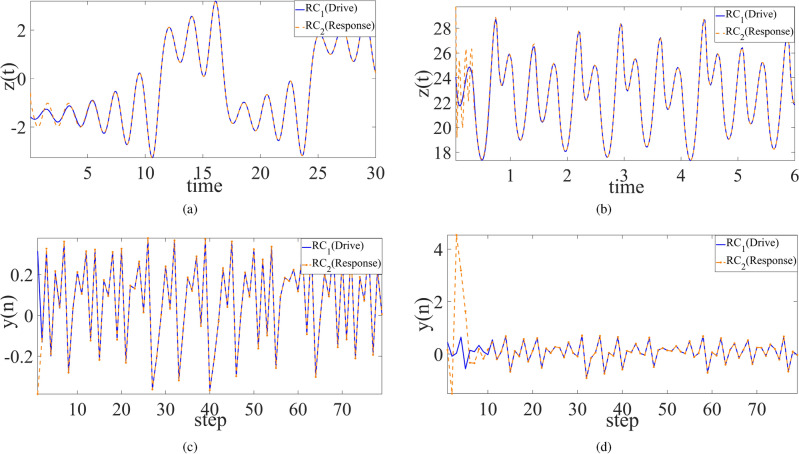
State variable evolution plots of synchronization between two RC systems trained from (**a**) Chua system, (**b**) PLUC system, (**c**) Hénon system, and (**d**) Ikeda system, respectively. Here the drive variable is the same as in Fig. 2.

### PC synchronization between chaotic systems and trained RC systems

Next, we use the proposed RC model based on SADE algorithm to train the RC systems for all the chaotic systems under consideration, and perform PC synchronization between the chaotic system and the trained RC system, between two trained RC systems, and between a chaotic system and two trained RC systems through the corresponding drive variables shown in Table 2.

**Figure 4:**
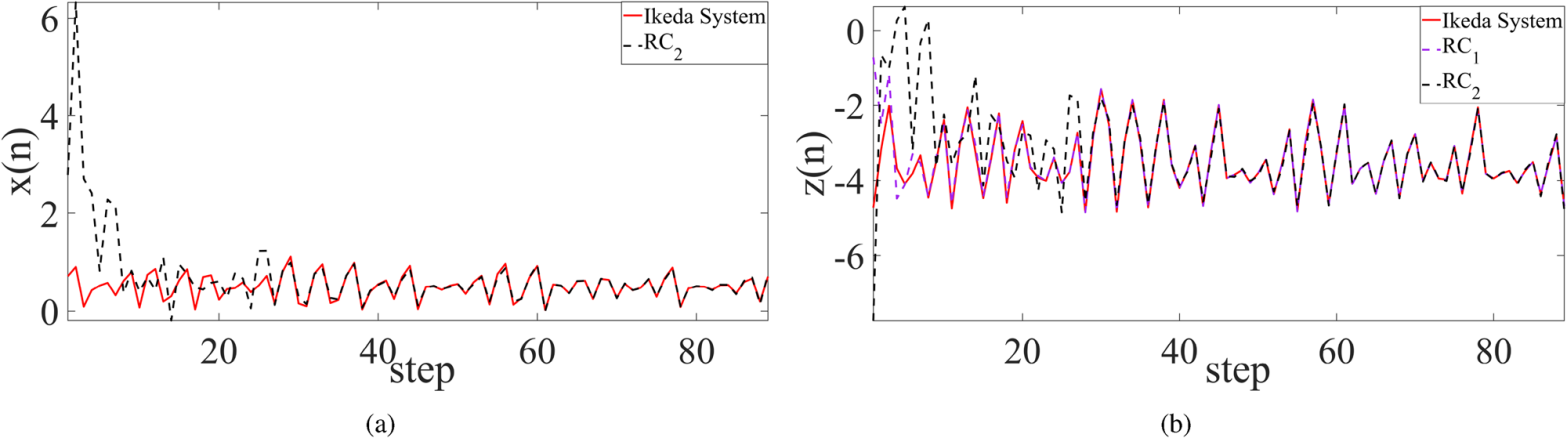
State variable evolution plots of cascade synchronization between (**a**) Ikeda system and the second trained RC system and (**b**) Ikeda system and the two trained RC systems.

Consequently, synchronization behavior of four non-smooth chaotic systems are shown in Figs. 2, 3, and 4 respectively for the cases between the chaotic system and its trained RC system, between two trained systems, and between Ikeda system and its two trained RC systems where the *x* component of Ikeda system is used to drive the first RC system, and the *y* component of the first RC system is used to drive the second RC system. The results demonstrate the good realization of PC synchronization, implying the availability of the improved RC model with SADE algorithm for non-smooth chaotic systems, of course as well as for smooth chaotic systems (synchronization evolution plots are not provided here).

**Figure 5:**
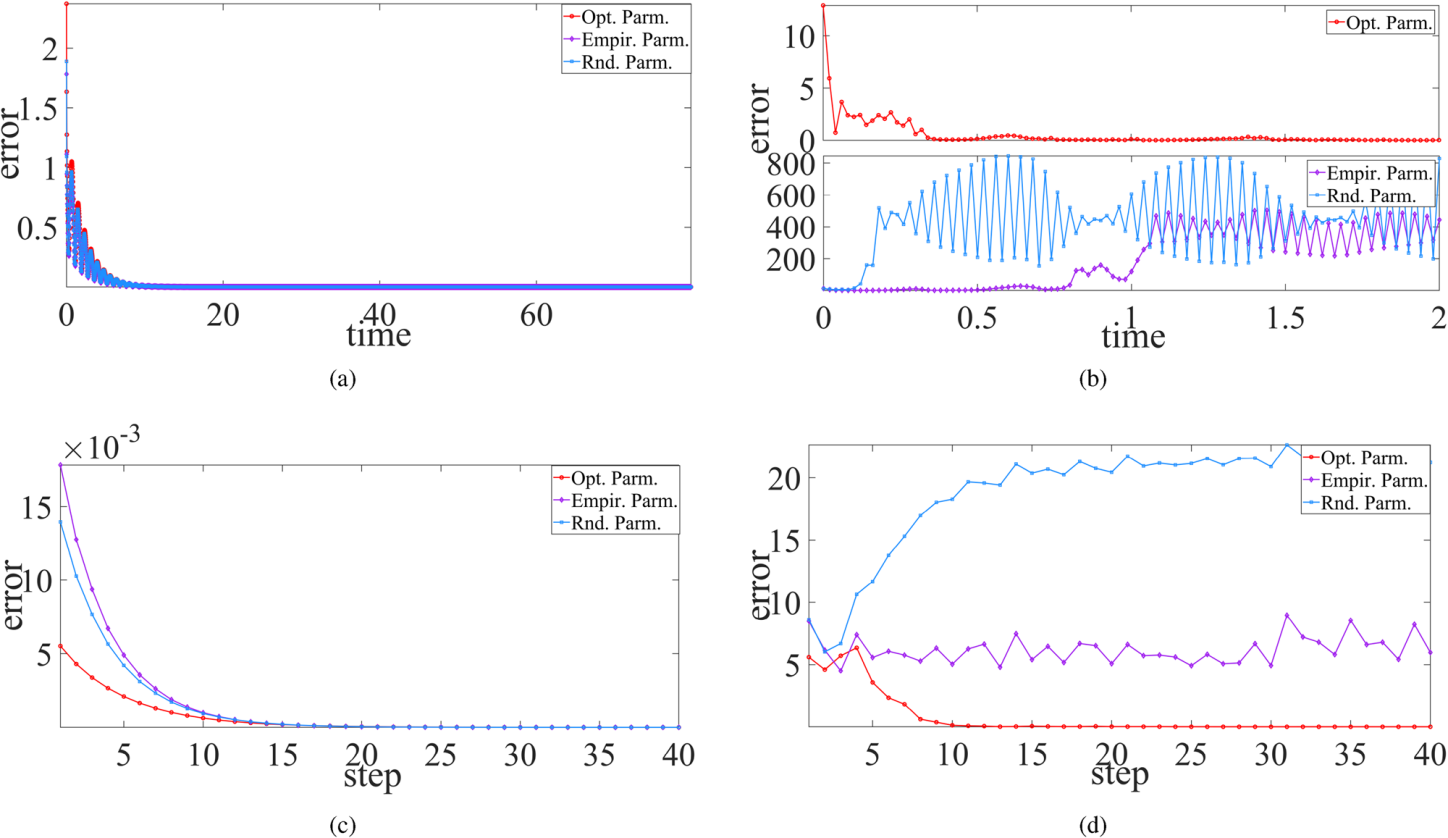
Synchronization error between the chaotic system and its trained RC systems with different types of parameters where RC models are trained from (**a**) Chua system, (**b**) PLUC system, (**c**) Hénon system, and (**d**) Ikeda system, respectively. Here the drive variable is the same as in Fig. 2.

**Figure 6:**
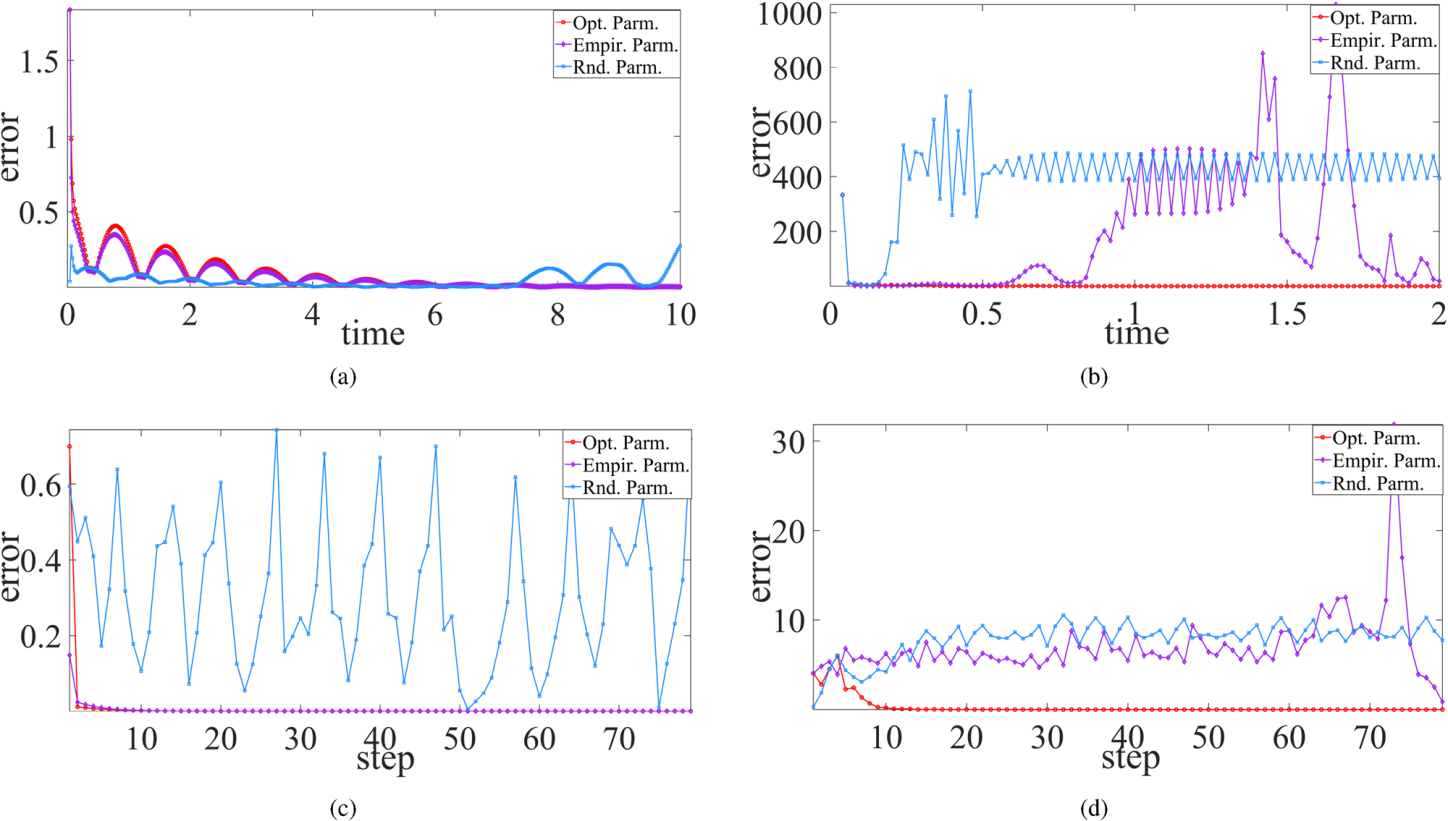
Synchronization error between two trained RC systems with different types of parameters where RC models are trained from (**a**) Chua system, (**b**) PLUC system, (**c**) Hénon system, and (**d**) Ikeda system, respectively. Here the drive variable is the same as in Fig. 2.

However, using empirical parameters and randomly-selected parameters, not all four non-smooth chaotic systems can be synchronized to their corresponding trained RC systems. As shown in Figs. 5 and 6, for PLUC system and Ikeda system, the synchronization error does not tend to zero as time goes, implying the failure of synchronization between the chaotic system and its trained RC system, as well as between two trained RC systems. Further, for Chua system, synchronization also fails between two trained RC systems with randomly-selected parameters (see Fig. 6a). These show the advantage of the proposed RC model with SADE algorithm and the importance of main parameters of ER network in RC model.

Interestingly, whatever using the empirical parameter or randomly-selected parameter, let alone the optimal parameter, the RC system trained from Hénon system works well, and it is able to be well synchronized with the original Hénon system as well as with its replicate RC system. A possible reason is the fact that the error $\Delta {\varvec{w}}\equiv 0$ in (8) as *x* is the drive variable.

## Discussion

In summary, for better learning synchronization behaviors of non-smooth chaotic systems, the paper has proposed an improved RC model based on SADE algorithm, which is capable of training up a set of optimal parameters in the reservoir, and thus of making a long-term prediction for chaotic systems. Also, it can work well for synchronization between the chaotic systems and well-trained RC systems. Significantly, the experiments have shown that the proposed method is effective not only for non-smooth chaotic systems, but for smooth chaotic systems. In other words, compared with the traditional RC, our RC based on SADE has a wider range of applications. Extensive simulations have shown that the proposed RC model has significant longer prediction time than the traditional RC model with empirical parameters or random parameters, and the trained RC systems are well synchronized to their original chaotic systems as well as the corresponding replicate RC systems whereas the traditional RC system can not yet work well.

Just because of the perfect prediction ability for chaotic systems, the well-trained RC system is more closely approximated to the original chaotic system, and thus it can successfully demonstrate synchronization behavior occurring in the drive-response chaotic system described in the second section. Theoretically, if the RC is sufficiently approximated to the original system, the variable driving the original drive-response system into synchronization is able to drive the trained RC system and its replica (or the original system) into synchronization. Conversely, the variable not driving the original one into synchronization should not drive two trained RC systems into synchronization.

Although the simple grid search approach may yield a as at least good result as the SADE approach in this work does, it spends far more time to find the optimal parameters than the SADE method. On the other hand, compared with the empirical or randomly-selected parameter approaches, the proposed method has significant advantage and performs well even for non-smooth chaotic systems, as shown in the above section. As a trade-off method, the proposed RC model with SADE algorithm is a good choice for training chaotic data and achieving PC synchronization. It may be applicable to many other chaotic systems including some complicated piecewise smooth chaotic systems only if avoiding the overfitting problem. Besides PC synchronization in the drive-response system where the coupling is unidirectional, the proposed RC model may be used to realize synchronization in the bi-directionally coupled non-smooth chaotic systems even the coupled networked chaotic systems.

## Methods

### Non-smooth chaotic systems

Here briefly reviews the used non-smooth chaotic systems, including piecewise smooth chaotic systems (Chua system and a piecewise linear unified chaotic system) and discrete chaotic systems (Hénon map and Ikeda map).

**Figure 7:**
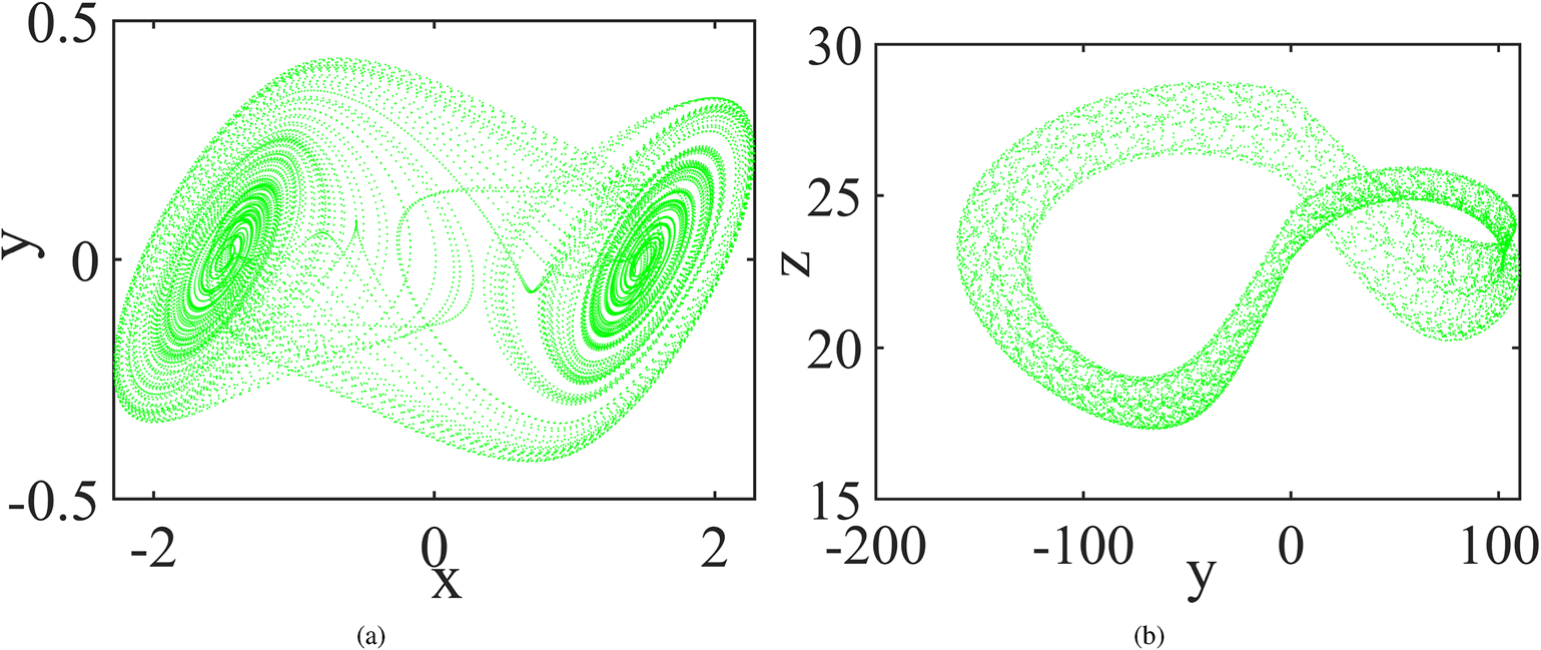
Attractors of Chua system (**a**) and PLUC system (**b**).

Chua System^39^:

1 $$\begin{aligned} {\left\{ \begin{array}{ll} \frac{dx}{dt}=10(-x+y-f(x)),\\ \frac{dy}{dt}=x-y+z, \\ \frac{dz}{dt}=-15y, \end{array}\right. } \end{aligned}$$

where $f(x)=\frac{2}{7}x-\frac{3}{14}(|x+1|-|x-1|)$. The phase diagram is shown in Fig. 7a.

Piecewise Linear Unified Chaotic (PLUC) System^40^:

2 $$\begin{aligned} {\left\{ \begin{array}{ll} \frac{dx}{dt}=35(y-x),\\ \frac{dy}{dt}=-7x-xz+28y, \\ \frac{dz}{dt}=sgn(y)x-3z, \end{array}\right. } \end{aligned}$$

where *sgn*(*y*) is the symbolic function, namely $sgn(y)=1$ when $y>0$, $sgn(y)=0$ when $y=0$ and $sgn(y)=-1$ otherwise. The phase diagram is shown in Fig. 7.

Hénon Map^41^:

3 $$\begin{aligned} {\left\{ \begin{array}{ll} x(n+1)=1-1.4x^2(n)+y(n),\\ y(n+1)=0.3x(n).\\ \end{array}\right. } \end{aligned}$$

Ikeda Map^42^:

4 $$\begin{aligned} {\left\{ \begin{array}{ll} x(n+1)=0.92+0.9(x(n)cos(z(n))-y(n)sin(z(n))),\\ y(n+1)=0.9(x(n)sin(z(n))+y(n)cos(z(n))),\\ z(n+1)=0.4-\frac{5.4}{1+x^2(n)+y^2(n)}. \end{array}\right. } \end{aligned}$$

**Figure 8:**
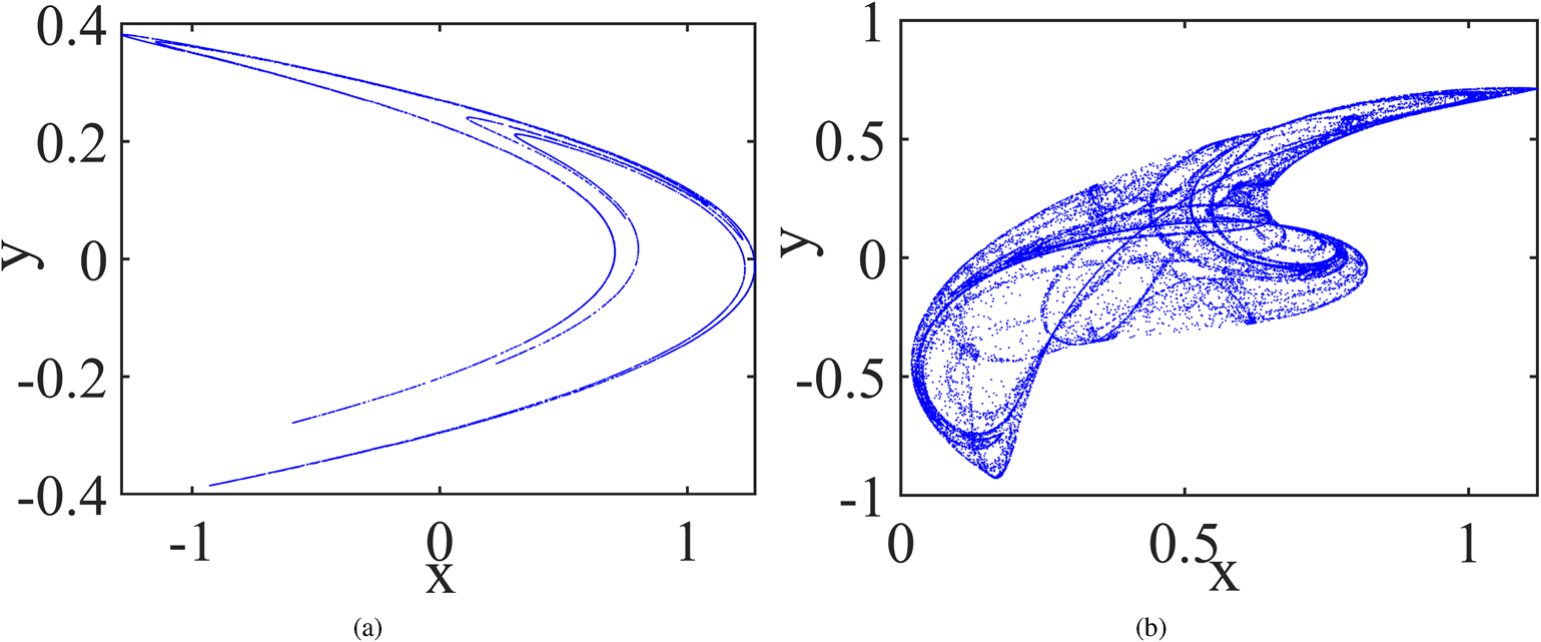
Attractors of Hénon map (**a**) and Ikeda map (**b**).

The phase diagrams of Hénon Map and Ikeda Map are shown in Fig. 8.

### PC synchronization principles

**Figure 9:**
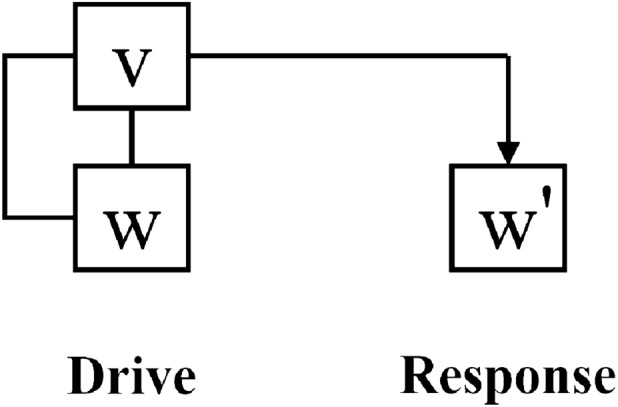
The schematic diagram for PC synchronization.

 In early years, Pecora and Carroll presented a variable substitution method^43,44^ for achieving synchronization between chaotic systems. The basic idea is to split the original system into two subsystems, copy one of the subsystems, and then replace the corresponding variable of the replicated subsystem with the variable (called the drive variable) of the other subsystem, and eventually realize synchronization between the replicated subsystem and the original system. The diagram is clearly shown in Fig. 9, and the mathematical model is described as follows.

For an *n*-dimensional chaotic system,

5 $$\begin{aligned} \frac{d{\varvec{u}}}{dt}=f({\varvec{u}}): {\left\{ \begin{array}{ll} \frac{du_1}{dt}=f_1(u_1,u_2, \ldots , u_n),\\ \qquad \vdots \\ \frac{du_n}{dt}=f_n(u_1,u_2,\ldots , u_n). \end{array}\right. } \end{aligned}$$

Divide arbitrarily it into two subsystems, for instance,

6 $$\begin{aligned} \frac{d{\varvec{v}}}{dt}=g({\varvec{v}},{\varvec{w}}): {\left\{ \begin{array}{ll} \frac{du_{1}}{dt}=f_{1}(u_1,u_2,\ldots ,u_n),\\ \qquad \vdots \\ \frac{du_{m}}{dt}=f_{m}(u_1,u_2,\ldots ,u_n), \end{array}\right. } \end{aligned}$$

and

7 $$\begin{aligned} \frac{d{\varvec{w}}}{dt}=h({\varvec{v}},{\varvec{w}}): {\left\{ \begin{array}{ll} \frac{du_{m+1}}{dt}=f_{m+1}(u_1,u_2,\ldots ,u_n),\\ \qquad \vdots \\ \frac{du_n}{dt}=f_{n}(u_1,u_2,\ldots ,u_n), \end{array}\right. } \end{aligned}$$

where $${\varvec{v}}=(u_{1},u_{2},\ldots ,u_{m})$$ and $${\varvec{w}}=(u_{m+1},u_{m+2},\ldots ,u_{n})$$.

Now create a new system $\frac{d{\varvec{w}}'}{dt}=h({\varvec{v}}',{\varvec{w}}')$ identical to the subsystem of $\frac{d{\varvec{w}}}{dt}=h({\varvec{v}},{\varvec{w}})$, substitute the variable $\varvec{v}'$ in the function $h({\varvec{v}}',{\varvec{w}}')$ with the corresponding variable ${\varvec{v}}$ (called the drive variable), and get the called response system $\frac{d{\varvec{w}}'}{dt}=h({\varvec{v}},{\varvec{w}}')$. Together with the drive system (5), it follows that

8 $$\begin{aligned} {\left\{ \begin{array}{ll} \frac{d{\varvec{v}}}{dt}=g({\varvec{v}},{\varvec{w}}),\\ \frac{d{\varvec{w}}}{dt}=h({\varvec{v}},{\varvec{w}}),\\ \frac{d{\varvec{w}}'}{dt}=h({\varvec{v}},{\varvec{w}}'). \end{array}\right. } \end{aligned}$$

The subsystems of ${\varvec{w}}$ and ${\varvec{w}}'$ is synchronized each other, called PC synchronization here, if the error $\Delta {\varvec{w}}={\varvec{w}}-{\varvec{w}}'\rightarrow 0$ as $t\rightarrow \infty$, which can be theoretically checked through the negative conditional Lyapunov exponent of the drive-response system (8). Through our checking, the system can achieve synchronization when the appropriate variables listed in Table 2 are selected as the drive variable.

**Table 2:** A list of driving and response variables for four non-smooth chaotic systems.

System	Drive	Response
Hénon	*x*	*y*
Ikeda	*x*	*y*, *z*
Chua	*x*	*y*, *z*
PLUC	*y*	*x*, *z*

In recent years, RC model is frequently applied and studied on account of its excellent predictive effect for nonlinear time series. As many researchers know, the reservoir consisting of ER random network structures plays a key role in this machine learning model, which can well characterize the connected neurons in the recurrent neural network, and is with strong dynamical characteristics.

In general, the parameters (network size *N*, connected probability *p* and network spectral radius *r*) of ER random networks in the reservoir are selected by experience, but the parameters have significant impact on the prediction effect, and therefore the inappropriate selection may lead to the poor prediction. In this section, we introduce a simulated annealing-based differential evolution (SADE) method for parameter selection, and present a reservoir computing system with SADE algorithm. Furtherly, we introduce how to realize PC synchronization via the presented reservoir computing model.

The reservoir computing system is composed of the input layer, the reservoir part, and the output layer. Denote ${\varvec{u}}(t)=(u_1(t),u_2(t),\ldots ,u_n(t))^T$ the input vector in the input layer, ${\varvec{r}}(t)=(r_1(t),r_2(t),\ldots ,r_m(t))^T$ the state vector in the reservoir, and ${\varvec{y}}(t)=(y_1(t),y_2(t),\ldots ,y_l(t))^T$ the output vector in the output layer. Then, the run of reservoir computers can be divided into three parts: Initialization, Training and Prediction, which are described as follows:

### Reservoir computing systems with SADE parameter selection algorithms

- Initialization: Generate the reservoir’s adjacency matrix *A* and the input weight matrix $W_{in}$. In general, *A* is a sparse ER random network matrix with the appropriate network size *N*, connected probability *p* and the spectral radius *r*, and its non-zero entries are randomly chosen from the uniform distribution of $[-1,1]$. The elements of matrix $W_{in}$ are randomly chosen from the uniform distribution of (-$\sigma , \sigma$).
- Training: Train the output weight matrix $W_{out}$ by using the training data set. Here we update the reservoir network state vector ${\varvec{r}}(t)$ according to the following iterative law:
  9 $$\begin{aligned} {\varvec{r}}(t+1)=(1-\alpha ){\varvec{r}}(t)+\alpha \tanh \left[ A{\varvec{r}}(t)+W_{in}\left( \begin{array}{c} b_{in}\\ {\varvec{u}}(t) \end{array}\right) \;\right] , \end{aligned}$$
  where $\alpha$ is the “leakage” rate limited in the interval of [0, 1], $\tanh$ is the hyperbolic tangent function, $b_{in}=1$ is the offset, and *A* and $W_{in}$ are the mentioned above adjacency matrix and input weights, respectively. To eliminate the transient state of the reservoir computer with the initial ${\varvec{r}}(0)$, the first $\tau$ time-steps states are removed out. Next, the state vector and the training data denoted $\{{\varvec{s}}(t)|t=\tau , \tau +1, \ldots , \tau +T\}$ are used to train the output $W_{out}$. Here the training data is taken as the input data $\{{\varvec{u}}(t)|t=\tau , \tau +1, \ldots , \tau +T\}$ where ${\varvec{u}}(t)$ is used to predict next forward ${\varvec{u}}(t+1)$. According to the reference^20^, the output weights $W_{out}$ can be calculated by
  10 $$\begin{aligned} W_{out}=YX^T(XX^T+\lambda I)^{-1}. \end{aligned}$$
  Where *I* is an identity matrix and $\lambda$ is a ridge regression parameter for avoiding overfitting. *X* is the matrix whose *t*-th column is $(b_{out},{\varvec{s}}(t),{\varvec{r}}(t))^T$, and *Y* is the one whose *t*-th column is ${\varvec{s}}(t+1)$.
- Prediction: With the trained output weights, the reservoir computing system based on Eqs. 9, 10, 11 can run autonomously, and use the current output vector ${\varvec{y}}(t)$ of (11) to predict ${\varvec{u}}(t+1)$ at the next moment, and so on.
  11 $$\begin{aligned} {\varvec{y}}(t)=W_{out}\left( \begin{array}{c} b_{out}\\ {\varvec{u}}(t)\\ {\varvec{r}}(t) \end{array}\right) . \end{aligned}$$

As mentioned in the beginning of this section, the number of nodes *N*, the connected probability *p* and the spectral radius *r* in ER random network of the reservoir, have a great impact on the prediction effect particularly for some chaotic systems. Our experiments have shown that the parameters selected by experience lead to poor prediction for some discrete chaotic systems and non-smooth chaotic systems.

**Figure 10:**
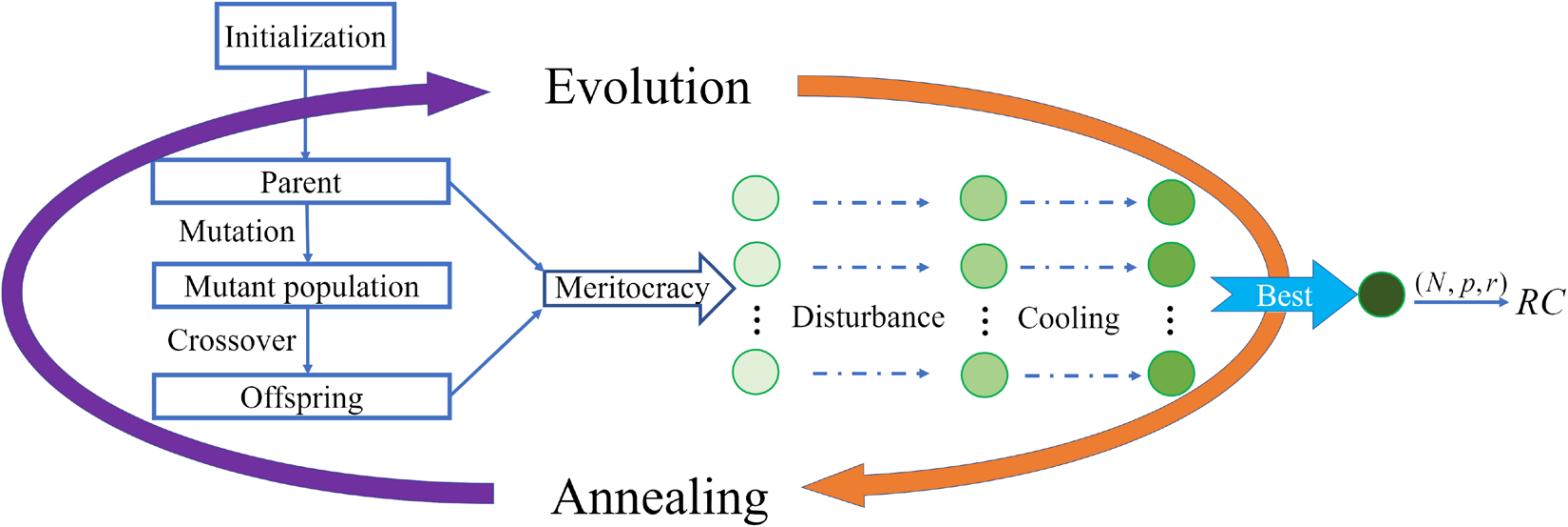
The schematic diagram for SADE algorithm, where each cycle includes evolution and annealing process. Each circle represents one individual with genes of *N*, *p*, and *r*, and different shades of color mean different groups. After some cycles of differential evolution and simulated annealing, the parameter corresponding to the best individual is chosen to generate the reservoir network of RC.

**Figure 11:**
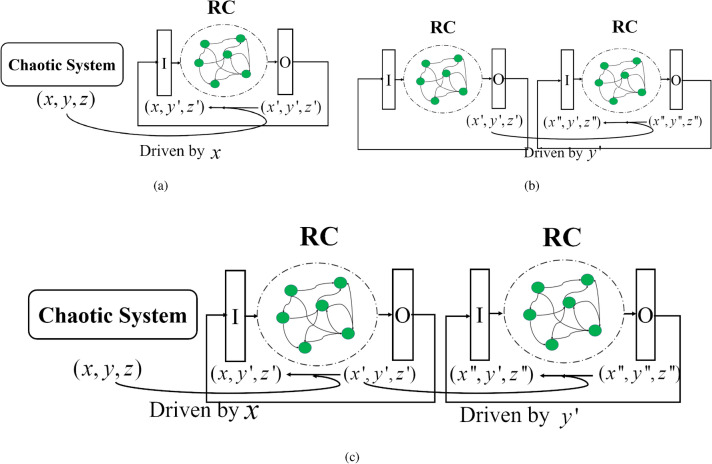
Diagrams for PC synchronization between different systems. (**a**) between chaotic system and its trained RC system, (**b**) between two trained RC systems, (**c**) cascade PC synchronization between chaotic system and its two trained RC systems.

For better prediction, we will introduce the simulated annealing-based differential evolution algorithm (SADE) to select the optimal parameters via which we train the reservoir computing system and then realize synchronization between trained reservoir computing systems.

The SADE algorithm is a combination of the differential evolution and the simulated annealing algorithms, where the former is a type of evolution algorithm with three steps of mutation, crossover and selection, and it aims to retain the better individual and eliminates the inferior individual until the fitness is met^45^. It can improve the population of feasible solutions and find the global optimal solution more quickly. The latter is an optimization algorithm imitating the physical process of solid annealing, and it can avoid being trapped into local optimal solution via the cooling procedure and the Metropolis rule. So, simulated annealing combined with different evolution algorithm may greatly improve the effect of optimizing parameters, and is employed to obtain the optimal parameters (*N*, *p*, *r*) for the adjacency matrix *A* in the reservoir computing system. The schematic diagram is illustrated in Fig. 10.

Based on the SADE algorithm, the parameter selection procedure includes four main phases. First, the initialization phase of population individuals, i.e., generating a three-dimensional parameter space (*N*, *p*, *r*). Second, the differential evolution phase for better solutions. Specifically, update the parameter setting for reservoir computing system, one can train the updated RC system through the chaotic time series, and then make the prediction via the trained RC system. If the fitness (here the mean square error is used) meets the requirement, the set of parameters is believed as the optimal parameters. Otherwise, mutation, crossover and selection operations are done to generate the new parameter individual, and then turns to the simulated annealing phase for further optimizing the parameter. Repeat the above operation until the fitness meets the requirement. Finally, it is the optimal parameters outputting phase for system prediction. Please see the Appendix for the detailed steps and procedure shown in shown in Fig. S1.

In general, compared with the differential evolution algorithm, the simulated annealing-based differential evolution algorithm has stronger global search ability, and provides the optimal parameters for better prediction. As shown in our extensive simulations (not given here), the prediction accuracy via SADE algorithm is least an order of magnitude higher than that via differential evolution algorithm whether for smooth chaotic systems or for non-smooth chaotic systems. Especially for non-smooth chaotic systems, the prediction accuracy can be improved up to three order of magnitude.

### Reservoir computing models for PC synchronization

Suppose ${\varvec{u}}(t+1)=F({\varvec{u}}(t))$ is the chaotic system to be learned, and ${\varvec{u}}'(t+1)={\hat{F}}({\varvec{u}}'(t))$ is the trained RC system after learning the chaotic system.

Take a three-dimensional chaotic system as an example, Fig. 11a illustrates the diagram of PC synchronization between the chaotic system and the trained RC system via the drive signal of *x* component, and the detailed implementation process is described as follows:

- Initiation. Generating the chaotic time series from ${\varvec{u}}(t+1)=F({\varvec{u}}(t))$ with the initial value ${\varvec{u}}_0=(x_0,y_0,z_0)^T$, and ${\varvec{u}}'(t)={\hat{F}}({\varvec{u}}'(t))$ with the initial value ${\varvec{u}}'_0=(x_0,y'_0,z'_0)^T$ where $y'_0$ and $z'_0$ different from $y_0$ and $z_0$ are randomly chosen.
- Substitution and update. Replacing $x'_t$ of the previous output $(x'_t,y'_t,z'_t)^T$ with $x_t$ of the chaotic data, getting the vector ${\varvec{u}}'(t+1)=(x_t,y'_t,z'_t)^T$, and then it is used as the next input vector to generate new iteration.
- Checking. If the errors $\Delta y(t)=|y'_t-y_t|$ and $\Delta z(t)=|z'_t-z_t|$ converge to zero as time, then the response system (the trained RC system) is synchronized with the drive system (the chaotic system) via the *x* component.

Similarly, we can create another replica of the trained RC system, and to realize the synchronization between two trained RC systems. The diagram is shown in Fig. 11b.

Suppose

12 $$\begin{aligned} {\varvec{u}}'(t+1)={\hat{F}}({\varvec{u}}'(t)), \end{aligned}$$

is the trained RC system where the input ${\varvec{u}}'=(x'_t,y'_t,z'_t)^T$, and create the replicate RC system denoted as

13 $$\begin{aligned} {\varvec{u}}''(t+1)={\hat{F}}({\varvec{u}}''(t)), \end{aligned}$$

where the input ${\varvec{u}}''=(x'_t,y''_t,z''_t)^T$ for which the initial value $y''_0$ and $z''_0$ are different from $y'_0$ and $z'_0$, and $x'_t$ is the drive variable come from the system (12).

According to the principle of PC synchronization, at each iteration, $x''_{t+1}$ compenont of the output ${\varvec{u}}''(t+1)=(x''_{t+1},y''_{t+1},z''_{t+1})^T$ in (13) is replaced with $x'_{t+1}$ of the output ${\varvec{u}}'(t+1)=(x'_{t+1},y'_{t+1},z'_{t+1})^T$ in (12), and the vector $(x'_{t+1},y''_{t+1},z''_{t+1})^T$ is used as the next input in (13) to perform the new iteration. Finally, using the error to check whether the synchronization between two RC systems is achieved or not.

For the cascade synchronization between a chaotic system and two RC systems shown in Fig. 11c, take *x* and *y* components as the drive variables, and suppose the chaotic system and two trained RC systems are described as

14a $$\begin{aligned} {\varvec{u}}(t+1)=F({\varvec{u}}(t)), \end{aligned}$$

14b $$\begin{aligned} {\varvec{u}}'(t+1)={\hat{F}}({\varvec{u}}'(t)), \end{aligned}$$

14c $$\begin{aligned} {\varvec{u}}''(t+1)={\hat{F}}({\varvec{u}}''(t)), \end{aligned}$$

where ${\varvec{u}}(t)=(x_t,y_t,z_t)^T$, ${\varvec{u}}'(t)=(x_t,y'_t,z'_t)^T$ and ${\varvec{u}}''(t)=(x''_t,y'_t,z''_t)^T$, and the initial values ${\varvec{u}}_0=(x_0,y_0,z_0)^T$, ${\varvec{u}}'_0=(x_0,y'_0,z'_0)^T$ and ${\varvec{u}}''_0=(x''_0,y'_0,z''_0)^T$. Here, the chaotic system (14a) is called the drive system, RC systems (14b) and (14c) are called the first and second response system, respectively.

At each new iteration, the vector $(x_{t+1},y'_{t+1},z'_{t+1})^T$ obtained by replacing $x'_{t+1}$ of the output ${\varvec{u}}'(t+1)=(x'_{t+1},y'_{t+1},z'_{t+1})^T$ in (14b) with $x_{t+1}$ of the output ${\varvec{u}}(t+1)=(x_{t+1},y_{t+1},z_{t+1})^T$ in (14a), is used as the input vector in (14b) for next iteration. Following up, the vector $(x''_{t+1},y'_{t+1},z''_{t+1})^T$ obtained by replacing $y''_{t+1}$ of the output ${\varvec{u}}''(t+1)=(x''_{t+1},y''_{t+1},z''_{t+1})^T$ in (14c) with $y'_{t+1}$ of the output ${\varvec{u}}'(t+1)=(x'_{t+1},y'_{t+1},z'_{t+1})^T$ in (14b), is used as the input vector in (14c) for next iteration. Finally, one can check the error between ${\varvec{u}}$, ${\varvec{u}}'$ and ${\varvec{u}}''$ for the cascade synchronization between the chaotic system and two response RC systems.

### Supplementary Information

Supplementary Information.

## Data Availability

All data generated and all programs used during the current study are available from the corresponding author on reasonable request.
