# Supplementary material for: Synchronization of non-smooth chaotic systems via an improved reservoir computing
Source: Sci Rep. 2024 Jan 2;14:229. doi: 10.1038/s41598-023-50690-4 (PMC10761703; doi:10.1038/s41598-023-50690-4)
Supplement: Supplementary file 1 — Supplementary Information. [file 41598_2023_50690_MOESM1_ESM.pdf]

Supplementary Materials for "Synchronization of non-smooth chaotic systems via an improved reservoir computing"

Guyue Wu, Longkun Tang*, Jianli Liang

Fujian Province University Key Laboratory of Computational Science,

School of Mathematical Science, Huaqiao University, Quanzhou 362021, China

1 The detailed SADE algorithm

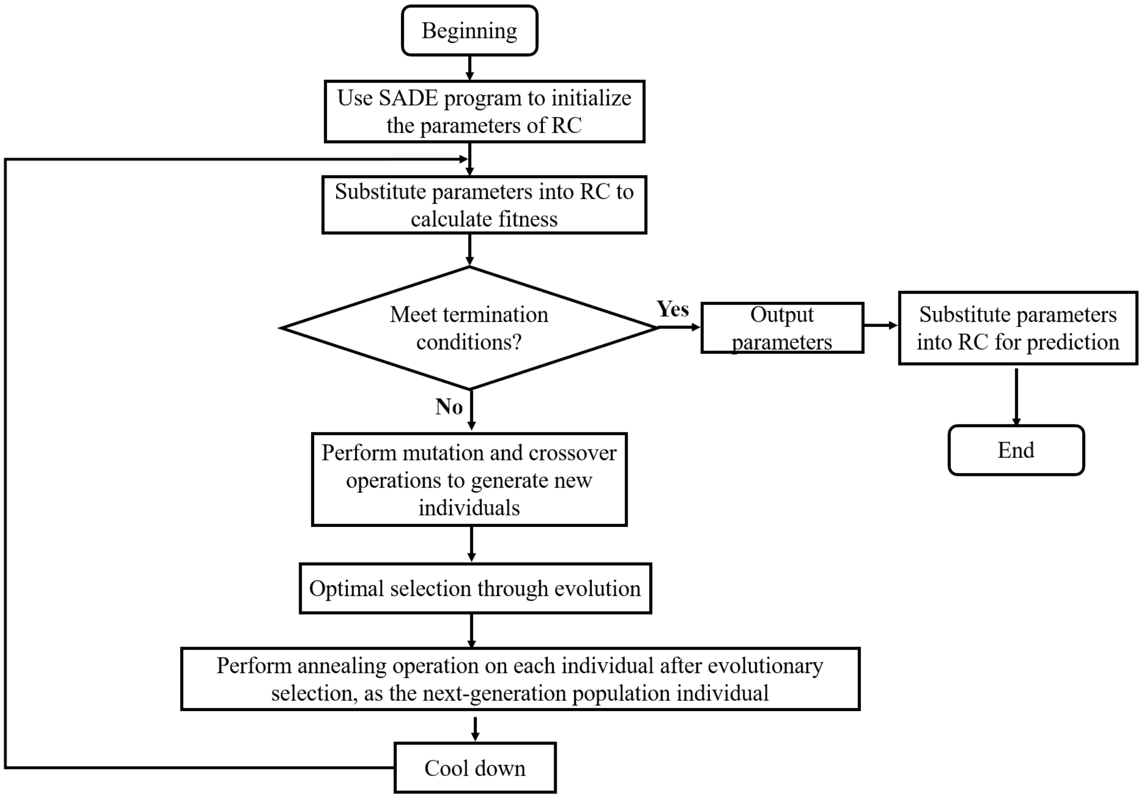

Fig S1. The flow chart of RC model with SADE algorithm.

For convenience, denote $A_i(g_{i_1}, g_{i_2}, g_{i_3})$ as the adjacency matrix of the i-th ER network of the reservoir where g_{i_1}, g_{i_2}, and g_{i_3} are three genes of individual A_i, corresponding to the parameters of N, p and r respectively. According to the flow chart shown in Fig. S1, the detailed process for parameter selection is described as follows:

- Initialization. Randomly generate a population $G = \{A_i(g_{i_1}, g_{i_2}, g_{i_3}), i = 1, 2, \cdots, NP\}$ with NP individuals where $g_{i_k} \in D_k\,(k = 1, 2, 3)$ is the k-th gene of the i-th individual A_i, and D_k is the value range of the k-th parameter. Here G is called the parent population.

- Mutation. At each time, we randomly select three individuals A_{i_1}, A_{i_2} and A_{i_3} from the parent population G, and use their genes to generate an descendant. Here, NP offspring individuals are generated in total, and the gene mutation rule is as below:

$$u_{i_k} = g_{i_{1_k}} + F_{i_{1_k}} r_0 (g_{i_{2_k}} - g_{i_{3_k}}), i = 1, 2, \cdots, NP,$$

$$u_{i_k} = g_{j_{1_k}} + F_{j_{1_k}} r_0 (g_{j_{2_k}} - g_{j_{3_k}}),\ j_1, j_2, j_3 \in \{1, 2 \cdots, NP\},$$

where $F_{i_{1_k}} = \min\{\frac{g_{i_{1_k}} - l_k}{d_k}, \frac{L_k - g_{i_{1_k}}}{d_k}\}$ is the mutation factor of the gene $g_{i_{1_k}}$, l_k and L_k are the lower and upper bound of its corresponding parameter value, respectively, $d_k = L_k - l_k\,(k = 1, 2, 3)$, and r_0 is a random number in the interval of $[0, 1]$ (the symbols below marked as 'r_1', 'r_2' and 'r_3' have the same meaning of 'r_0' but their values are different from r_0 due to randomness).

- Crossover. Cross over each mutated individual, and form a new individual $\tilde{A}_i$ and its gene $\tilde{g}_{i_k}$ is generated according to the following formula:

$$\tilde{g}_{i_k} = \begin{cases} u_{i_k}, & Cr < r_1, k \neq randi(3), \\ g_{i_k}, & \text{otherwise}, \end{cases} \tag{1}$$

where Cr is the crossover factor, and $randi(3)$ is a random integer in the interval of $[1, 3]$. The newly formed population after the crossover is denoted as $\tilde{G} = \{\tilde{A}_i(\tilde{g}_{i_1}, \tilde{g}_{i_2}, \tilde{g}_{i_3}), i = 1, 2, \cdots, NP\}$.

- Retain outstanding individuals in the population and then produce the annealed particle swarm. Specifically, we compare the fitness values of A_i and $\tilde{A}_i$, and retain the better individual denoted as B_i, namely,

$$B_i = \begin{cases} A_i, & Fit(A_i) < Fit(\tilde{A}_i), \\ \tilde{A}_i, & \text{otherwise}, \end{cases} \tag{2}$$

where $Fit(\cdot)$ is the fitness function (here take the mean square error of the prediction). Consequently, a particle group $P = \{B_i(v_{i_1}, v_{i_2}, v_{i_3}), i = 1, 2, \cdots, NP\}$ is produced.

- Perform annealing operation on P. First, one exert a suited perturbation on each individual in P to produce the new particles:

$$\tilde{v}_{i_k} = \begin{cases} v_{i_k} + \beta^n r_2 R_k, & \frac{r_k}{d_k} < 0.1, \\ v_{i_k} - \beta^n r_2 r_k, & \frac{r_k}{d_k} > 0.9, \\ v_{i_k} + sgn(r_0 - 0.5)\beta^n r_0 \min\{r_k, R_k\}, & \text{otherwise}. \end{cases} \tag{3}$$

where $r_k = v_{i_k} - l_k$, $R_k = L_k - v_{i_k}$, β is the annealing coefficient, and n means the n-th generation evolution. Next, it accepts the new particle state $\tilde{B}_i(\tilde{v}_{i_1}, \tilde{v}_{i_2}, \tilde{v}_{i_3})$ or not according to the Metropolis rule:

$$M_i = \begin{cases} B_i, & \text{condition}\, Q \\ \tilde{B}_i, & \text{otherwise} \end{cases} \tag{4}$$

where condition Q: "$Fit(B_i) < Fit(\tilde{B}_i)$" or "$Fit(B_i) \geq Fit(\tilde{B}_i)$ and $r_3 < e^{-\frac{\Delta E}{T}}$". After annealing, the population $M = \{M_i, i = 1, 2, \cdots, NP\}$ can be obtained, and then the current temperature T is updated by $T_{n+1} = \beta T_n$.

- Calculate the fitness of each particle in M and find the best individual. If there exists the individuals whose fitness values meet the preset requirement, then the best individual M_i (the individual with minimum fitness value) in all the individuals meeting the requirement is outputted, and the corresponding matrix is used as the adjacency matrix of the reservoir. Otherwise, replace group G in Step 2 with group M, then repeat Step 2–Step 5 until there exist the individuals whose fitness values meet the preset requirement, or the number of iterations reaches the preset value.

2 Experiment results on delayed Hénon system

This paper focuses on non-smooth chaotic systems and smooth chaotic systems without delays, as the supplement of smooth chaotic systems without delays, as well as the other one of Hénon system pair, a delayed smooth Hénon system is used to show the effectiveness and superiority of the proposed RC model with SADE method. Here the delayed Hénon system [1] is described as follows:

$$\begin{cases} \frac{dx}{dt} = -\frac{1}{\sigma}x(t) + \frac{1}{\sigma} - \frac{a}{\sigma}x^2(t-\tau_1) + \frac{1}{\sigma}y(t-\tau_1), \\ \frac{dy}{dt} = -\frac{1}{\sigma} + \frac{b}{\sigma}x(t-\tau_2). \end{cases} \tag{5}$$

Where $a = 1.4$, $b = 0.3$ and $\sigma = 1$, and τ_1 and τ_2 are the time delay.

As shown in Fig. S2, the RC model based on SADE method performs well in the prediction (Fig. S2(a), (b), (c), (d)) and PC synchronization (Fig. S2(e), (f)), and does far better than the empirical and randomly-selected parameters methods. For the prediction, the data from RC models with empirical or randomly-selected parameters is going divergent. Similar as discussed in [2], the time delay may has influence on the performance of RC models, and the performance in the case of $\tau_1 = 3, \tau_2 = 13$ (Fig. S2(a), (b)) is better than that in the case of $\tau_1 = 3, \tau_2 = 36.5$ (Fig. S2(c), (d)).

For PC synchronization, the proposed RC model with optimal parameters also has significantly better performance than RC models with empirical or randomly-selected parameters, see Fig. S2(e), (f). By the way, the variable substitution can pulls the data of RC model with empirical parameters or randomly-selected parameters into a normal-bound trajectory.

References

[1] Tomás Caraballo, Renato Colucci, and Luca Guerrini. Dynamics of a continuous hénon model. *Math. Methods Appl. Sci.*, 41(10):3934–3954, 2018.

[2] Herbert Jaeger. The “echo state” approach to analysing and training recurrent neural networks. In *GMD−German National Research Institute for Computer Science*, page 148, 2001.

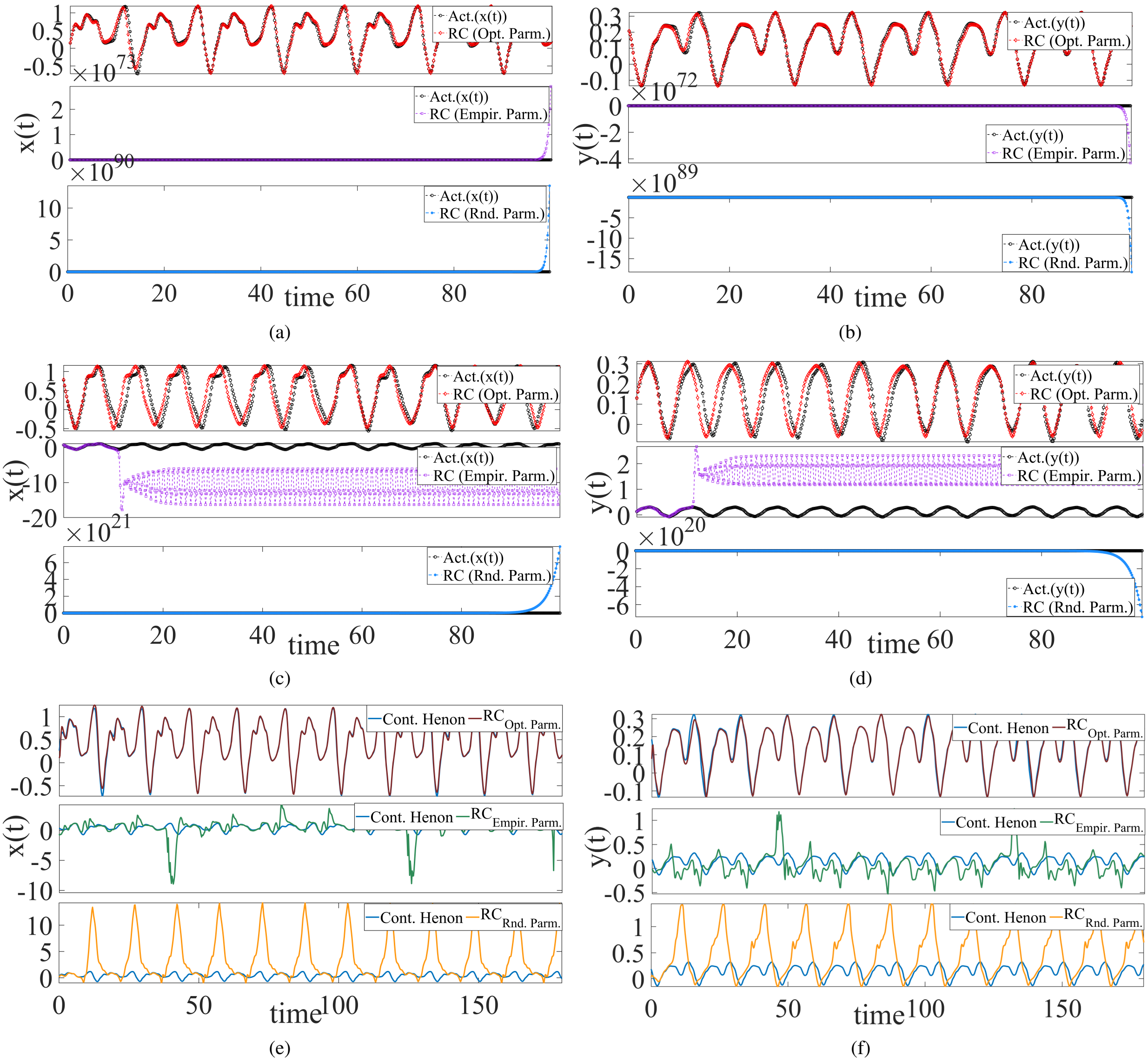

Fig S2. The performance of prediction and synchronization for delayed Hénon systems in using RC models with optimal, empirical, randomly-selected parameters, respectively. Prediction of Hénon system with $\tau_1 = 3$, $\tau_2 = 13$ (a) (b), and with $\tau_1 = 3$, $\tau_2 = 36.5$ (c),(d). (e) (f) PC synchronization between Hénon system ($\tau_1 = 3$, $\tau_2 = 13$) and RC models.
